# Frequency of Hypogonadism and Erectile Dysfunction in Type-II Diabetic Patients

**DOI:** 10.7759/cureus.2654

**Published:** 2018-05-19

**Authors:** Samsam Mushtaq, Khurshid Khan, Saleeha Abid, Amina Umer, Tabish Raza

**Affiliations:** 1 Department of Medicine, Jinnah Hospital, Allama Iqbal Medical College, Lahore, PAK; 2 Jinnah Allama Iqbal Institute of Diabetes and Endocrinology (jaide), Jinnah Hospital, Allama Iqbal Medical College, Lahore, PAK; 3 Department of Obstetrics and Gynaecology, Jinnah Hospital, Allama Iqbal Medical College, Lahore, PAK; 4 Department of Medicine and Endocrinology, Jinnah Hospital, Allama Iqbal Medical College, Lahore, PAK

**Keywords:** diabetes mellitus, hypogonadism, erectile dysfunction, frequency, testosterone deficiency

## Abstract

Introduction: The persistent state of hyperglycemia in diabetes mellitus predisposes diabetic patients to suffer from neuropathy, vasculopathy, and endocrinological changes. Hypogonadism and erectile dysfunction are commonly observed in diabetic patients secondary to androgen deficiency. In the developing world, patients usually under-report their sexual dysfunction. We conducted this study to determine the frequency of hypogonadism and erectile dysfunction and the associated risk factors in type-II diabetic male patients presenting in the outpatient department of a tertiary care hospital in Pakistan.

Methods: This was an observational, cross-sectional hospital-based study conducted at Jinnah Allama Iqbal Institute of Diabetes and Endocrinology (JAIDE), Allama Iqbal Medical College/Jinnah Hospital, Lahore, Pakistan from April 2017 to October 2017. One hundred and sixty type-II diabetic patients meeting the inclusion criteria were enrolled in the study after informed consent. All patients were given the International Index of Erectile Function (IIEF) questionnaire to determine the severity of erectile dysfunction. Patients were tested for serum total testosterone levels and hypogonadism labeled if the serum testosterone level came out to be less than 8 nmol/L with or without symptoms of hypogonadism or with a testosterone level of 8-12 nmol/L and symptoms of hypogonadism.

Results: The mean age of the patients was 51.2 ± 11.5 years (range: 31 – 60 years). The mean duration of type-II diabetes mellitus was 8.3 ± 5.1 years. The frequency of erectile dysfunction was found to be 62.5%. Mild erectile dysfunction was seen in 19 patients (11.9%), mild to moderate in 15 patients (9.4%), moderate in 42 patients (26.2%), and severe in 24 patients (15.0%) with an IIEF score of 17-21, 12-16, 8-11, and 1-7, respectively. More severe erectile dysfunction was seen in patients with a prolonged history of diabetes (p-value <0.0001). The mean testosterone level was 18.1 + 8.4 (range: 0.31-38.1) nmol/L. Based on our criteria, hypogonadism was seen in 61 patients (38.1%) with 29 (18.1%) and 32 (20.0%) suffering from severe and mild forms of hypogonadism, respectively. Forty percent of the patients with erectile dysfunction suffered from some form of hypogonadism with subnormal testosterone levels. The difference in the testosterone level of patients with and without erectile dysfunction was statistically significant (p-value: 0.0001).

Conclusion: Patients suffering from type-II diabetes mellitus had a significantly greater frequency of erectile dysfunction and hypogonadism. Diabetic patients should be counseled and treated for these issues to improve their quality of life, especially in under-developed countries where sexual health problems are seldom reported.

## Introduction

Diabetes is a metabolic syndrome characterized by hyperglycemia resulting from defects in insulin secretion, insulin action, or both [1]. The persistent state of hyperglycemia has serious effects on virtually every body system, including but not limited to neuropathy, vasculopathy, as well as endocrinological changes, which all together lead to grave effects on the patient’s quality of life [2]. Androgen deficiency has recently been identified as the dark side of type-II diabetes in male patients, leading to hypogonadism and erectile dysfunction [2]. It was ignored previously mainly by the patients themselves, as they associated it with increasing age and took it as a normal phenomenon [3]. However, an increased understanding of the diabetes pathophysiology has brought this to light, and there are studies that report that a considerable proportion of diabetic patients suffer these complications [4].

The reported frequency of hypogonadism and erectile dysfunction varies from 11.8% to 97.2% in different populations [2-4]. A possible explanation for this variation among studies can be the population differences, patient’s compliance and diabetic control, and the overall sexual health of the patients [5-7]. The topic has little attention in the recent medical literature from our country. In the developing world, sex is a taboo and type-II diabetic patients find it embarrassing to ask the doctor about such issues, particularly in front of attendants [8]. Knowledge of the magnitude of this problem will enable the doctors to anticipate and ask leading questions to the patient to identify and treat these complications, which have serious social impacts on the patient’s quality of life.

## Materials and methods

This was a cross-sectional study conducted at the department of endocrinology, Jinnah Hospital, Lahore, from April 2017 to October 2017. The study was conducted following the principles of good clinical practice, as laid down in the Declaration of Helsinki, after approval of its synopsis by the ethical review board of Allama Iqbal Medical College, Lahore. A sample size of 160 cases was calculated using a 95% confidence level and a 5% margin of error and taking the expected frequency of hypogonadism to be 11.8% in patients with Type-II diabetic patients [5]. Patients aged between 20 and 60 years diagnosed with type-II diabetes mellitus in the past 24 months were recruited in the study using non-probability, consecutive sampling. Patients with a history of a coronary event or procedure (myocardial infarction, unstable angina, coronary artery bypass surgery, or coronary angioplasty) in the previous four weeks, patients with liver disease (serum bilirubin ≥ 1.2 mg/dl), renal disease (serum creatinine levels ≥ 1.5 mg/dl), alcoholics (≥ 500 ml/week of alcohol for ≥ 6 months period), and patients with a history of hypothyroidism were excluded from the study.

One hundred and sixty type-II diabetic patients presenting in the outpatient department of endocrinology, Jinnah Hospital, Lahore, meeting the inclusion criteria were enrolled in the study. Written informed consent was obtained from each participant. The patients' age, gender, and duration of diabetes were noted. All the patients were given the International Index of Erectile Function (IIEF) questionnaire to determine the severity of erectile dysfunction. Patients were tested for serum total testosterone levels from the department of pathology, Jinnah Hospital, Lahore, and hypogonadism labeled if the serum testosterone level came out to be less than 8 nmol/L with or without symptoms of hypogonadism or with a testosterone level of 8-12 nmol/L and symptoms of hypogonadism.

All the collected data were analyzed using Statistical Package for Social Sciences (SPSS) version 20.0 (IBM Statistics Incorporated, Chicago, IL, USA). Numerical variables like age, duration of diabetes, IIEF score, and serum total testosterone level were presented by mean ± SD. Whereas, categorical variables like hypogonadism and erectile dysfunction were presented as frequency and percentages. Data were stratified for the age and duration of diabetes to address effect modifiers. The post-stratification chi-square test was applied to test for statistical significance, taking p ≤ 0.05 as significant.

## Results

There were 160 type-II diabetic male patients in the study with a mean age of 51.2 ± 11.5 years (range: 31 – 60 years). The mean duration of type-II diabetes mellitus was 8.3 ± 5.1 years. The mean values of HbA1c, body mass index (BMI), and waist circumference are given in Table 1.

**Table 1:** Demographic Characteristics of the Study Population BMI: body mass index

Study variable	Mean ± S.D.	Range
Age (years)	51.2 ± 11.5	31 – 60
Duration of diabetes (years)	8.3 ± 5.1	1 – 25
HbA1c (%)	9.2 ± 1.54	5.9 – 12.3
BMI (Kg/m^2^)	26.4 ± 6.1	16.2 – 41.8
Waist circumference (cm)	90.2 ± 12.3	68 – 134

The overall frequency of erectile dysfunction came out to be 62.5% in the study population. A breakdown of the severity of the erectile dysfunction showed that it was mild in 19 patients (11.9%), mild to moderate in 15 patients (9.4%), moderate in 42 patients (26.2%), and severe in 24 patients (15.0%), with an IIEF score of 17-21, 12-16, 8-11, and 1-7, respectively (Table 2). Sixty patients (37.5%) did not have any form of erectile dysfunction with an IIEF score of more than 22 (Table 2).

**Table 2:** Frequency of Erectile Dysfunction in the Study Population IIEF: International Index of Erectile Function

Erectile Dysfunction	IIEF Score	Frequency (n)	Percentage (%)
None	22 – 25	60	37.5
Mild	17 – 21	19	11.9
Mild to Moderate	12 – 16	15	9.4
Moderate	8 – 11	42	26.2
Severe	1 – 7	24	15.0
Total	1 – 25	160	100.0

The results of this study showed that the prevalence of erectile dysfunction increased with an increasing duration of diabetes mellitus (p-value <0.001). The prevalence of erectile dysfunction ranged from 26.0% to above 90.0% in patients with diabetes for less than five years to those with diabetes for more than 12 years, respectively. A greater severity of erectile dysfunction was seen in patients with a longer history of diabetes (p-value <0.0001). The severity of erectile dysfunction with a deteriorating score on IIEF was observed with a prolonged history of diabetes. The severe form of erectile dysfunction with an IIEF score between 1 and 7 was observed in 0.0% and 66.7% of the patients with a history of diabetes for the last five and 15 years, respectively (p-value <0.001) (Table 3).

**Table 3:** Relationship of Duration of Diabetes with Severity of Erectile Dysfunction IIEF: International Index of Erectile Function; ED: erectile dysfunction

Duration of Diabetes (years)	Severity of Erectile Dysfunction (n, %)					Total (n, %)
	No ED	Mild ED	Mild to Moderate	Moderate	Severe	
IIEF Score	22 – 25	17 – 21	12 – 16	8 – 11	1 – 7	
< 5	40 (72.7)	5 (9.1)	2 (3.6)	8 (14.6)	0 (0.0)	55 (100.0)
6 – 10	12 (27.9)	10 (23.3)	6 (13.9)	11 (25.6)	4 (9.3)	43 (100.0)
11 – 15	7 (19.4)	3 (8.3)	5 (14.0)	13 (36.1)	8 (22.2)	36 (100.0)
> 15	1 (3.8)	1 (3.8)	2 (7.7)	10 (38.6)	12 (46.1)	26 (100.0)
Total	60 (37.5)	19 (11.9)	15 (9.4)	42 (26.2)	24 (15.2)	160 (100.0)
Chi-square value: 90.12 p-value: 0.001						

The mean testosterone level was 18.1 + 8.4 nmol/L and the range was 0.31-38.1 nmol/L. The distribution of total testosterone levels showed levels of 9.8, nmol/L, 16.1 nmol/L, and 23.1 nmol/L at the 25th, 50th, and 75th percentiles, respectively. Based on our criteria described in the methodology section, the overall hypogonadism was seen in 61 patients (38.1%) with 29 (18.1%) and 32 (20.0%) suffering from a severe and a mild form of hypogonadism, respectively. The maximum amount of testosterone was observed in the sixth decade of age with p-value <0.001. Forty percent of the patients with erectile dysfunction suffered from some form of hypogonadism with subnormal testosterone levels. The difference in the testosterone levels of patients with and without erectile dysfunction was statistically significant (p-value: 0.0001). However, the differences in the other study variables like BMI, waist circumference, and duration of diabetes were not significantly different between the patients with and without erectile dysfunction (p-value > 0.05) (Table 4).

**Table 4:** Comparison of Various Study Variables Between Patients With and Without Hypogonadism Body mass index: BMI; WC: waist circumference

Study Variable	Eugonadism	Mild Hypogonadism	Severe Hypogonadism	P-value
Total testosterone level (nmol/L)	21.6 ± 3.9	11.2 ± 4.3	4.9 ± 2.8	< 0.0001
Age (years)	50.8 ± 11.2	51.4 ± 12.1	52.1 ± 12.4	0.185
HbA1c (%)	6.2 ± 2.1	9.2 ± 2.0	11.7 ± 2.3	< 0.0001
BMI (kg/m^2^)	26.2 ± 5.9	26.0 ± 6.0	27.1 ± 6.3	0.435
WC (cm)	90.6 ± 10.8	91.1 ± 9.9	91.6 ± 9.1	0.866

## Discussion

The primary objective of the study to find the frequency of erectile dysfunction and hypogonadism in type-II diabetic male patients was successfully met. There are many theories on hypogonadism and erectile dysfunction in diabetes, with causes ranging from vasculopathy to neuropathy and endocrinopathy [4]. Some studies have even claimed psychogenic causes to be one of the most challenging treatment dilemmas for patients with diabetic erectile dysfunction [5]. However, it is unknown at present whether diabetes brings about any changes in the personal effects that result in erectile dysfunction or there are any other underlying factors that cause changes in the psychology of the patients [6]. But, scientific literature that explains the underlying vasculopathy appears to be a more effective way of causing erectile dysfunction in such patients. As the research on finding the causes of diabetes mellitus and erectile dysfunction continues, so do the studies to determine the actual prevalence and associated risk factors of erectile and endocrine dysfunction in diabetes.

Much of earlier published research has reported a frequency of diabetes-associated hypogonadism and erectile dysfunction. The reports on the prevalence of erectile dysfunction range from 61% to 67% [7-10]. The results of our study match these results, as the frequency of erectile dysfunction came out to be 67.5% in our study population. Corona et al. reported that diabetes mellitus was associated with the typical symptoms of hypogonadism like decreased libido, decreased sexual attempts, and a high rate of depression. Besides, the size of the testes and the levels of luteinizing hormone were also decreased, which suggested a central nervous system basis of the disease. Similarly, the penile systemic vascular flow was significantly reduced in diabetic males [3].

The variations in the reported studies can be explained on the basis of differences in the study designs, study populations, erectile dysfunction evaluating instruments, and various cut-off limits for gonadal hormones. It is worth mentioning here that with the introduction of various anti-diabetic drugs and the aggressive treatment of diabetes, one might expect a lower prevalence of erectile dysfunction in diabetes [11-12]. A high rate of diabetes-related hypogonadism and erectile dysfunction in our population shows that the diabetic control of our population was not strict and required more efforts to reach optimal glucose levels in our patients.

Another factor that needs consideration in our part of the world is a lack of education and the inability to fill out the proforma by the patients themselves. The patients were asked to answer all the questions in the questionnaire but a majority could not due to a lack of education. So, the investigators asked the questions and recorded the answers in the response sheets. We anticipate that this might have underestimated the problem since very few people are open and true about their underlying sexual health in developing countries [8]. Erectile dysfunction is considered to be a loss of male power and not many people would like to declare and acknowledge it in our part of the world [13]. If the participants could fill the questionnaires themselves, we expect to see a greater prevalence of erectile dysfunction in our population.

The results of the present study also showed that with an increasing duration of diabetes mellitus, the prevalence of erectile dysfunction also increased. The same trend has been shown by earlier studies, which showed that as the duration of diabetes increased, so did the prevalence of erectile dysfunction in the population [14-16]. The underlying mechanism may be a severe vascular compromise in the form of atherosclerosis in the vascular corporal tissues, causing an inability to initiate, attain, and/or maintain penile erection [17-19].

Inquires about sexual health and its related symptoms demand a lot of empathy and rapport-building with the patients so that they can express all their hidden symptoms and concerns freely with the treating physician [16]. In a culture where sexual health is considered taboo, a lot of responsibility lies on endocrinologists to educate the patients about sexual health so that can be treated for any underlying diabetes-related underlying erectile dysfunction and hypogonadism [18,20]. We recommend future studies based on an even larger sample size and taking into account various quality of life and sexual health indices in diabetic patients.

## Conclusions

Hypogonadism and erectile dysfunction are very common clinical features of patients suffering from long-standing diabetes mellitus. Clinicians should be aware of these under-reported pathologies related to diabetes and act professionally, maintaining confidentiality and privacy, to let patients express their concerns on these matters. If identified early, these conditions can be successfully treated, leading to an improved quality of life in diabetic patients.
